# Supplementary figures and images for: Single-Cell RNA Sequencing Unravels Upregulation of Immune Cell Crosstalk in Relapsed Pediatric Ependymoma
Source: Front Immunol. 2022 Jun 30;13:903246. doi: 10.3389/fimmu.2022.903246 (PMC9281506; doi:10.3389/fimmu.2022.903246)

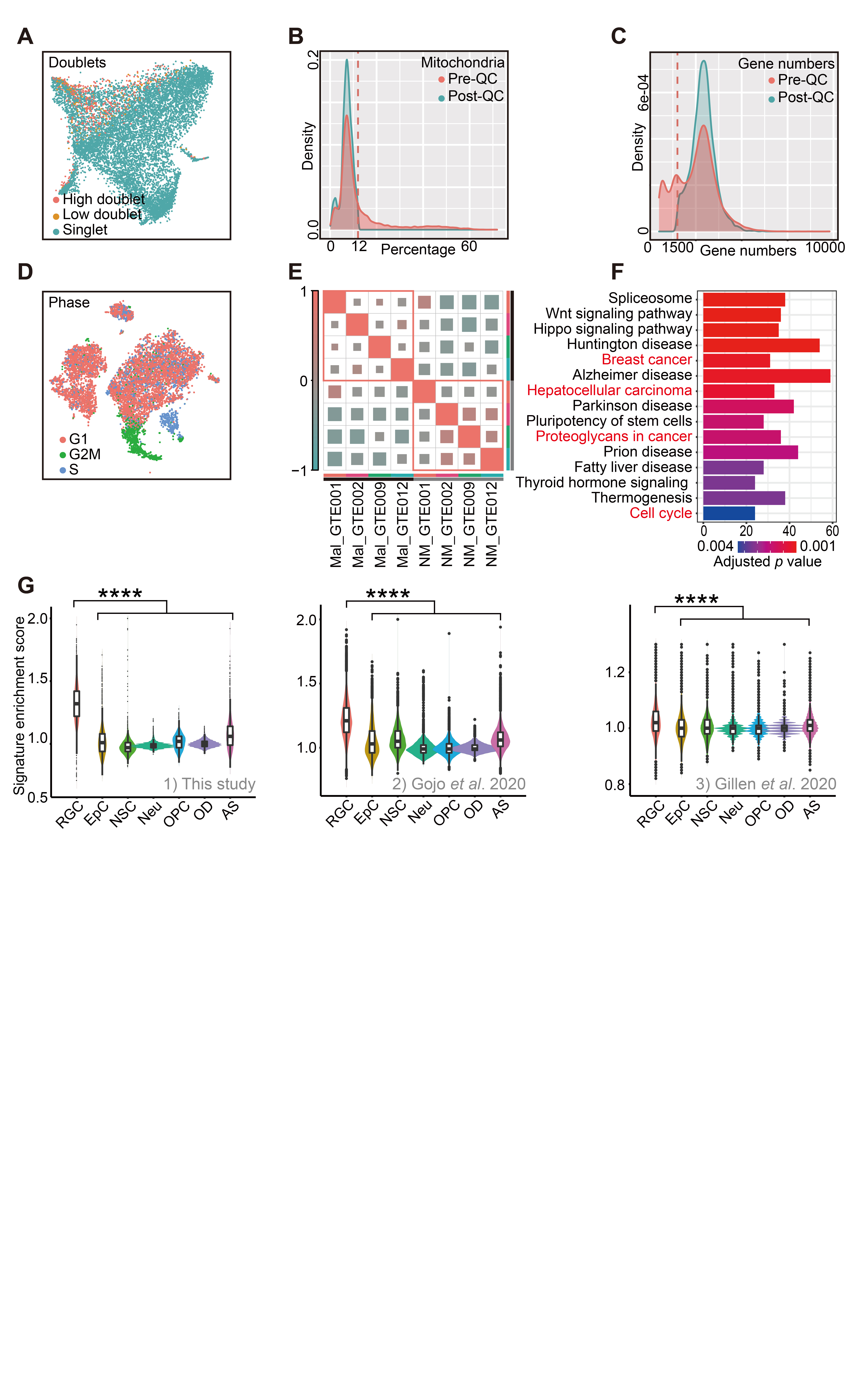

Supplement: Supplementary Figure 1 — Quality Control of scRNA-Seq Analysis of Human EPN. (A) Doublets identified by Doublet Finder presented on tSNE reduction. High doublet cells are filtered out. (B) Density plot of mitochondria percentage of cells in sample GTE009. Cells with more than 5% of mitochondria percentage are filtered out. (C) Density plot of captured gene numbers of cells in sample GTE009. Cells with less than 1500 transcripts are filtered. (D) Cell cycle phases of cells in sample GTE009 re-calculated after quality control presented on tSNE reduction. (E) Correlation plot of transcriptome of malignant tumor cells (Mal) and non-malignant cells (NM) among samples (GTE001, GTE002, GTE009, and GTE012). (F) KEGG analysis on DEGs of malignant tumor cells compared to non-malignant cells of four merged samples (GTE001, GTE002, GTE009, and GTE012). (G) Enrichment of RGC signatures in malignant tumor cells compared to other cells types using single-cell transcriptomes from 1) this study, 2) EPN (5) and 3) childhood EPN (6) (one-way ANOVA analysis; p value < 0.0001). [file Image_1.jpeg]

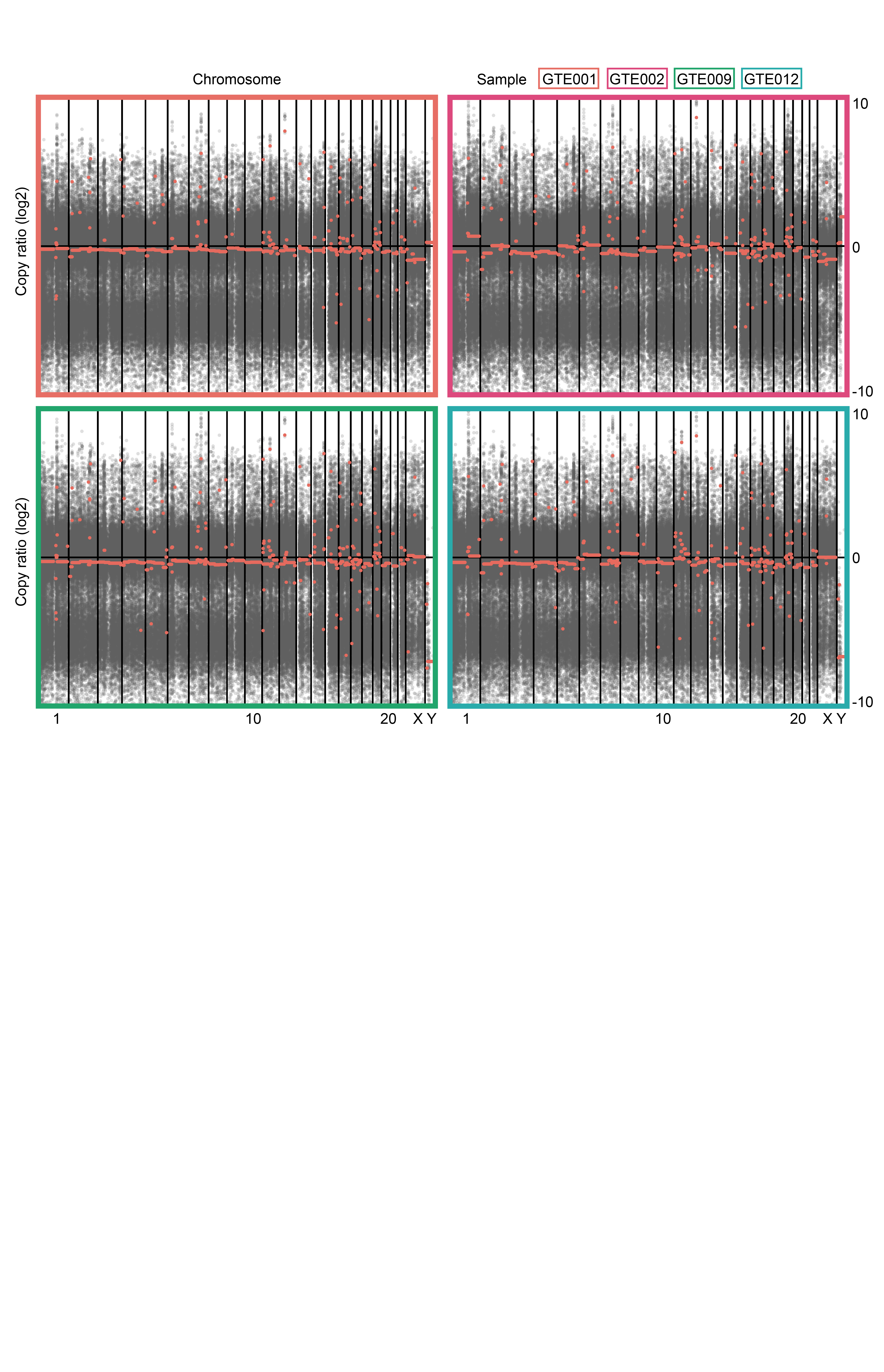

Supplement: Supplementary Figure 2 — CNV Analysis of Whole-exome Sequencing. CNV heatmap of whole-exon sequence data labeled by samples. [file Image_2.jpeg]

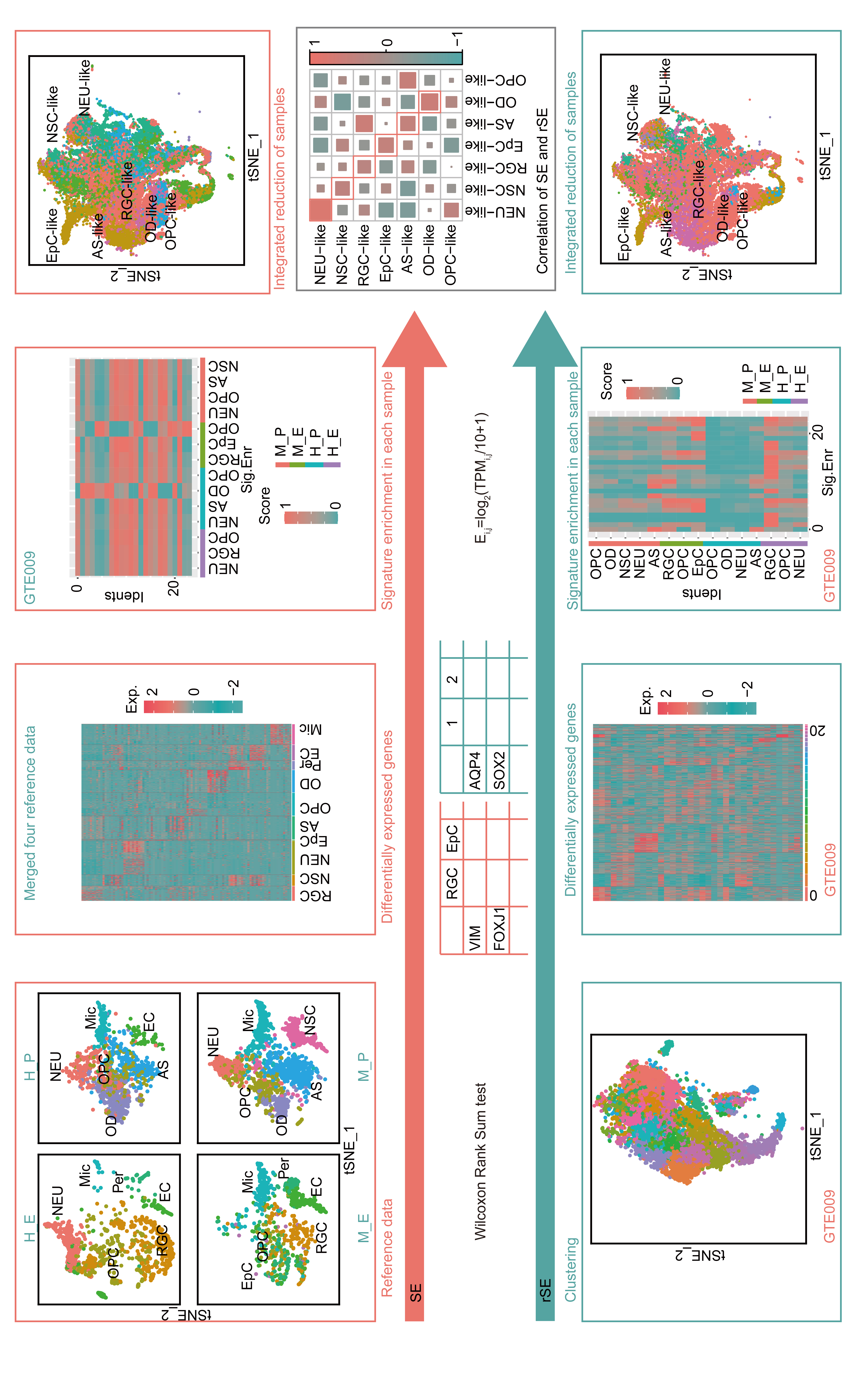

Supplement: Supplementary Figure 3 — Workflow of Cell Type Classification. Top: Schematic for cell type classification by signature enrichment (SE). The DEGs of different cell populations are obtained from published transcriptomic datasets of human and rodent embryonic and adult cortex (details see Supplementary Table 2 ) and used as signatures to distinguish cell types. All unknown cells are then clustered at high resolution to obtain multiple clusters, and the highest signature enrichment score in each cluster is designated as the cell type identity for these clusters. Bottom: Schematic for cell type classification by reversed signature enrichment (rSE). In the reciprocal analysis pipeline, unknown cells are first clustered at high resolution to obtain multiple clusters and the DEGs of all clusters are calculated and used as signatures to distinguish cell types. The signatures are then compared with published transcriptomic datasets of human and rodent embryonic and adult cortex (details see Supplementary Table 2 ), and the highest signature enrichment score is assigned as the name for the unknown cluster. The correlation result of SE- and rSE-determined cell types by ‘cor’ function of stats package in R v3.6.3 confirms high correlation across the two analysis pipelines. [file Image_3.jpeg]

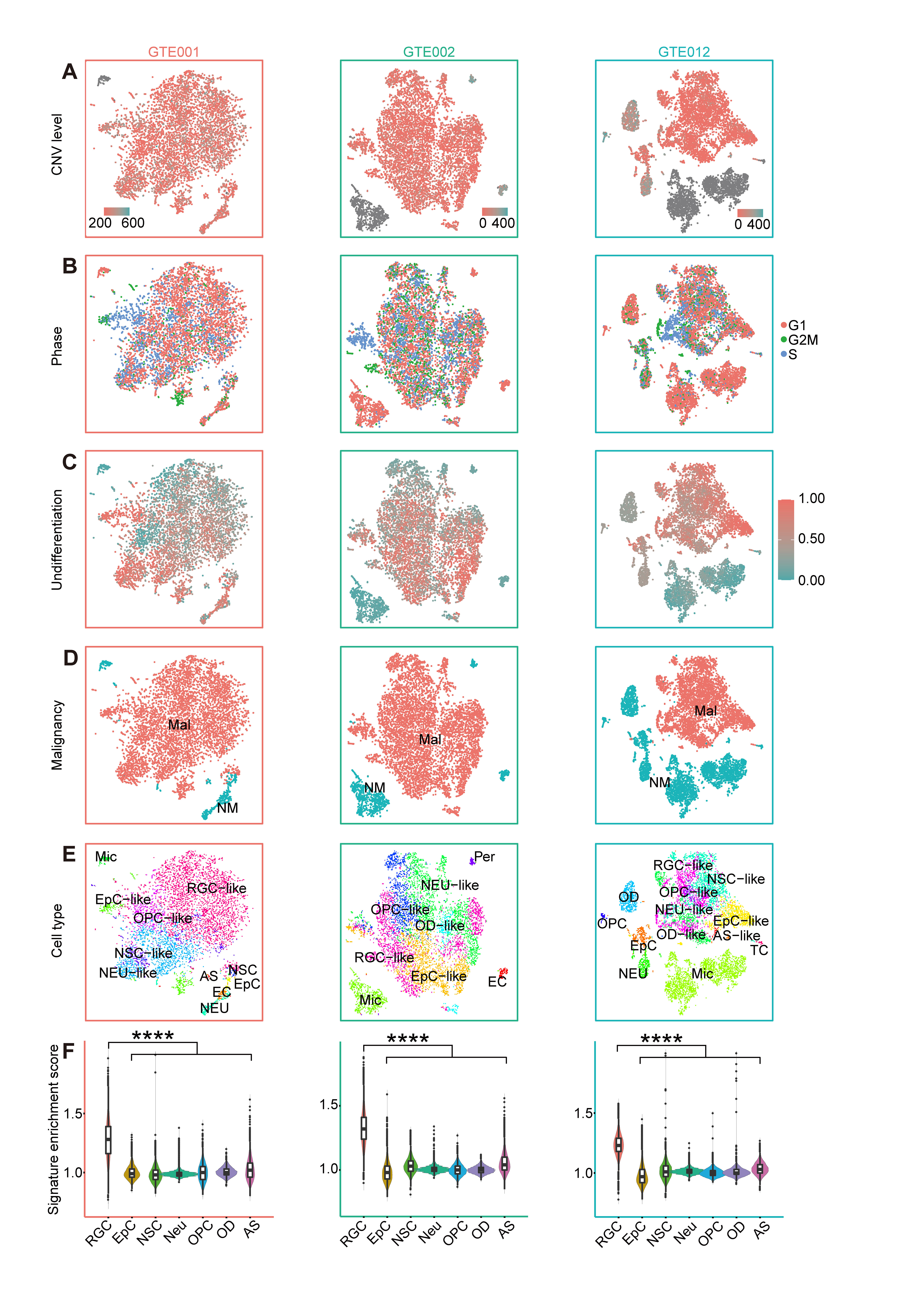

Supplement: Supplementary Figure 4 — Additional scRNA-Seq Analysis of Human EPN samples. (A) CNV score calculated by modified inferCNV of samples (GTE001, GTE002, and GTE012) presented on tSNE reduction. (B) Cell cycle phases in cellular level of samples (GTE001, GTE002, and GTE012) presented on tSNE reduction. (C) Undifferentiated score calculated by CytoTRACE of samples (GTE001, GTE002, and GTE012) presented on tSNE reduction. (D) Classified non-malignant cells and malignant tumor cells of samples (GTE001, GTE002, and GTE012) presented on tSNE reduction. (E) Annotated clusters of samples (GTE001, GTE002, and GTE012) presented on tSNE reduction with unbiased visualization by SCUBI (12). (F) Enrichment of signatures in malignant tumor cells compared to other cell types in samples (GTE001, GTE002, and GTE012; one-way ANOVA analysis; p value < 0.0001). See also Supplementary Table 1 . [file Image_4.jpeg]

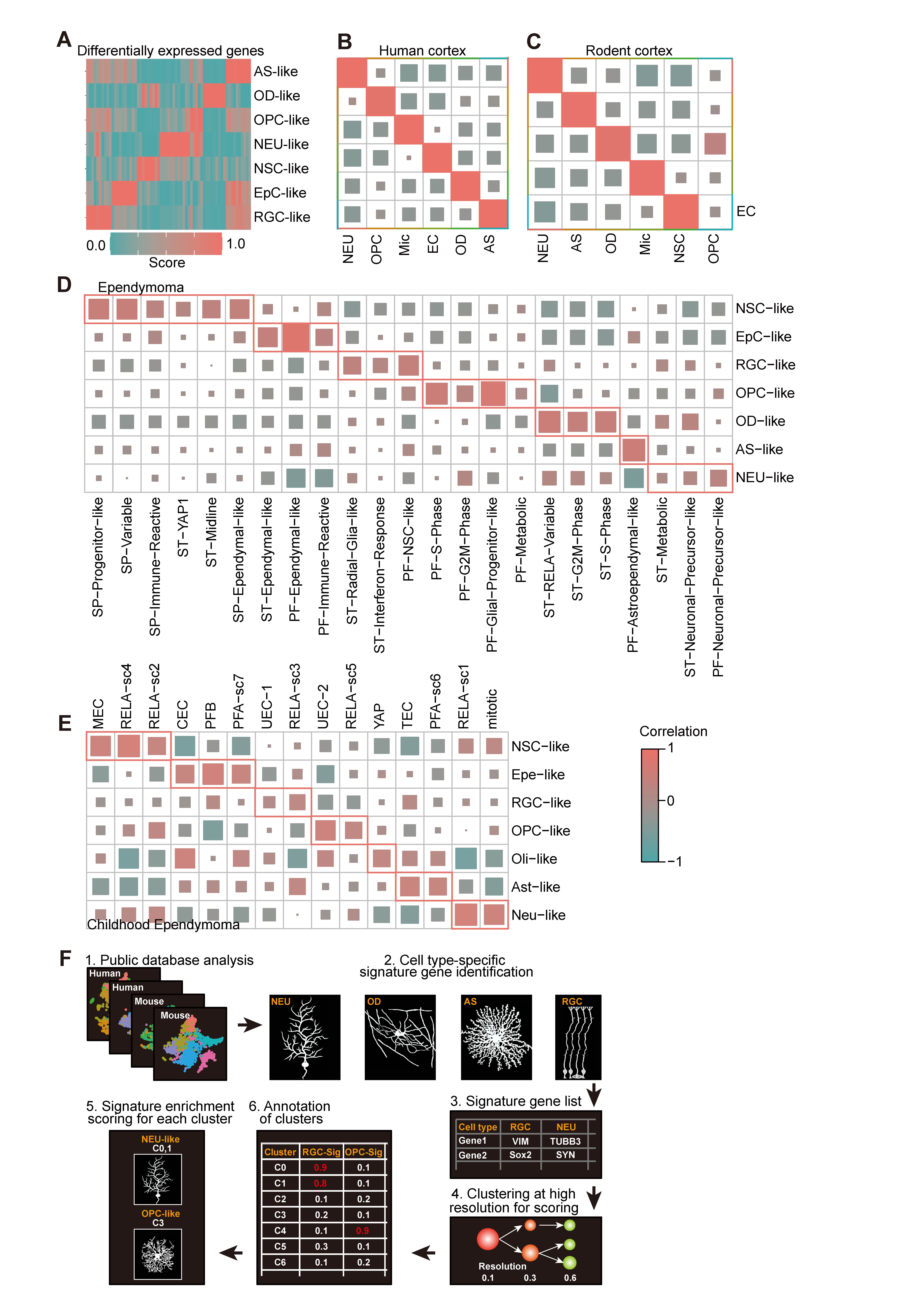

Supplement: Supplementary Figure 5 — EPN cell type classification validation. (A) Heatmap of DEGs in annotated clusters of the sample GTE009. (B–E), Correlation of cell types classified by method of signature enrichment (rows) and that of original cell types determined by original authors [B: human cortex (58), C: rodent cortex (63), D: ependymoma (5), and E: childhood ependymoma (6)]. (F) Workflow of cell-type classification. Signature markers genes are obtained from public transcriptome databases of human and rodent cortex and used for cell-type assignment and cluster annotation. [file Image_5.jpeg]

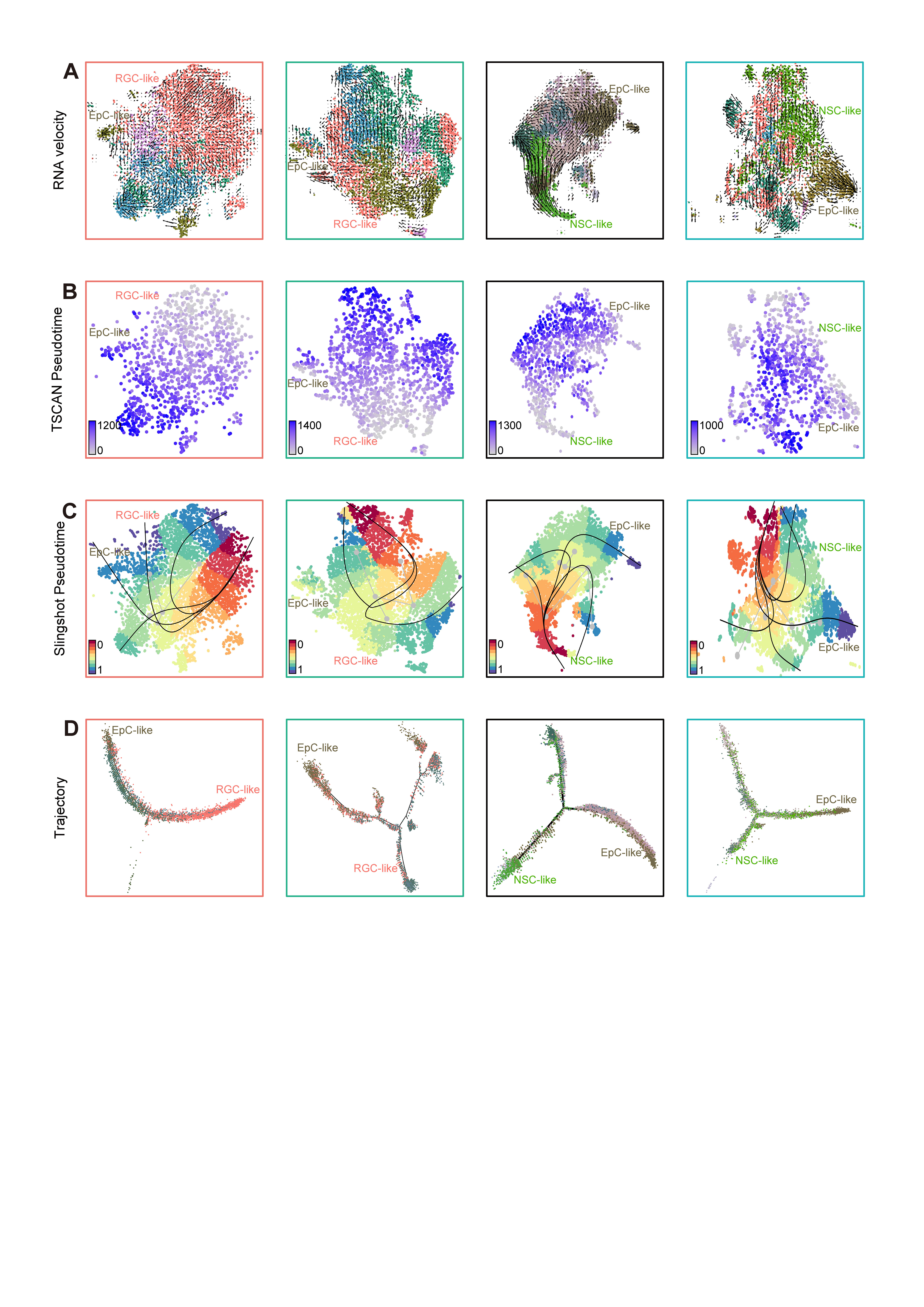

Supplement: Supplementary Figure 6 — Trajectory and pseudotime analysis. The columns are samples from patients (from left to right: GTE001, GTE002, GTE009, and GTE012). (A) RNA velocity inferred by Velocyto and scVelo of malignant tumor cells presented on tSNE reduction and colored by cell types. (B, C) Pseudo-time reconstruction and evaluation by TSCAN (41) (B) and by Slingshot (42) (C). (D) Differentiation trajectory inferred by Monocle of malignant tumor cells. [file Image_6.jpeg]

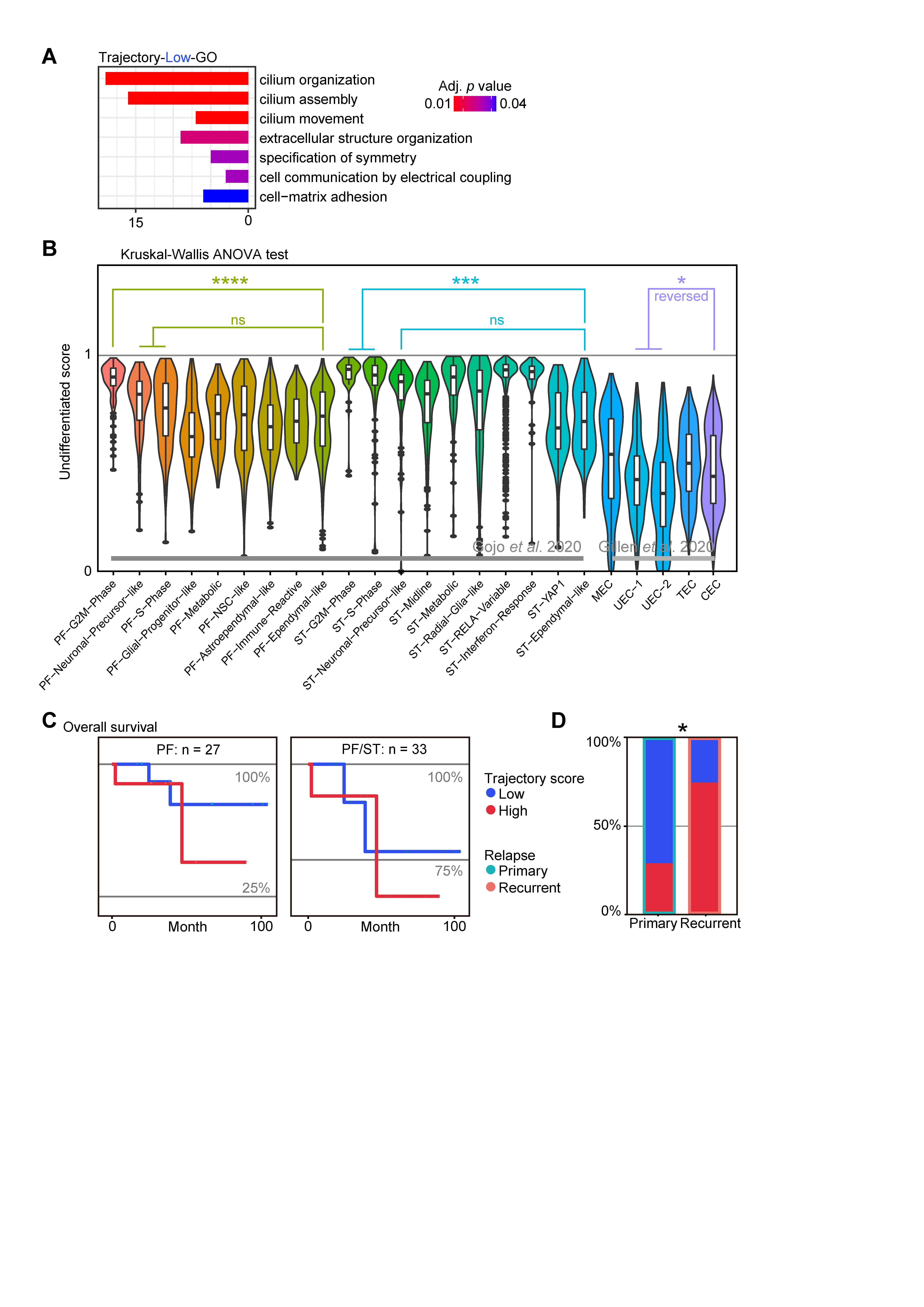

Supplement: Supplementary Figure 7 — Additional trajectory score analysis of EPN. (A) Gene ontology analysis of upregulated genes in patients with low trajectory score compared to patients with high trajectory score in published ependymoma scRNA-seq data (5, 6). (B) Undifferentiated score analysis on published scRNA-seq datasets of ependymoma (5, 6) (Kruskal-Wallis test). (C) Overall survival analysis on the trajectory score. A p value of 0.39 and 0.72 were obtained from the difference between two groups of trajectory score of patients (separated by the mean of trajectory score), with a trend of worse survival in patients with high trajectory score. The high and low group were separated by the mean of trajectory score on published scRNA-seq data (5, 6). (D) Histogram showing percentage of cells with high and low trajectory score and outlined by subclone annotation in samples from primary and recurrent patients. Permutation test shown significant compositional difference between primary and recurrent samples (p value = 0.01241; asymptotic two-sample Fisher-Pitman permutation test). *p < 0.05; **P < 0.01; ***p < 0.001; ****p < 0.0001 [file Image_7.jpeg]

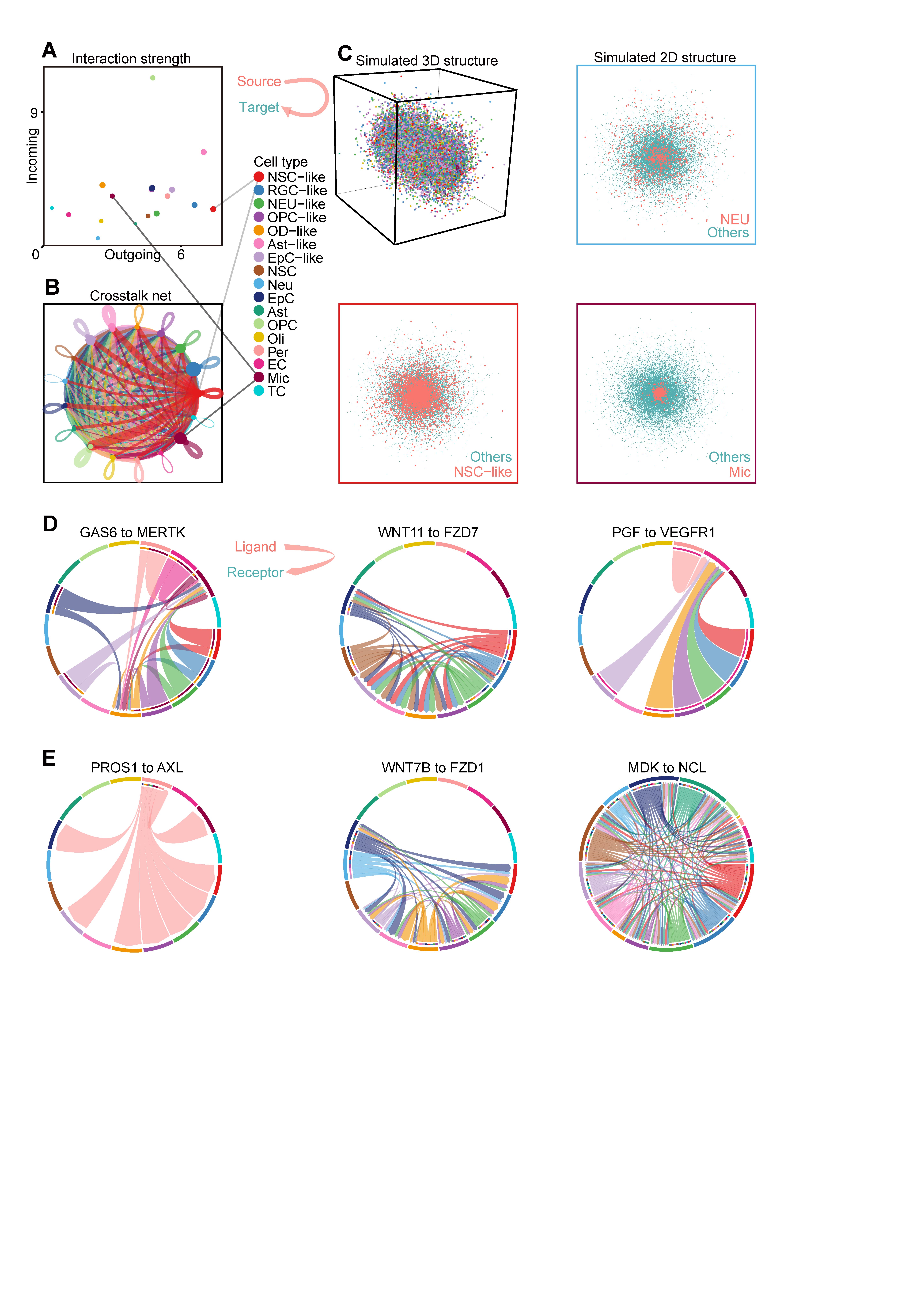

Supplement: Supplementary Figure 8 — Additional crosstalk analysis of EPN. (A) Interaction strength between cell types profiled in EPN samples inferred by CellChat. (B) Crosstalk net analyzed by CellChat. Individual lines represent the crosstalk from source to target cells. (C) Simulated 3D spatial structure of all cells and 2D angle of the simulated spatial structure by CSOMAP (34) of Mic, NSC-like cells, and NEU cells respectively, colored by pink (cell type of interest) and blue (other cell types). (D, E) Circle plots of ligands and receptors with higher expression in recurrent samples than that in primary samples. Lines represent the crosstalk between specific ligands and colors represent the cell type origin for each interaction [file Image_8.jpeg]

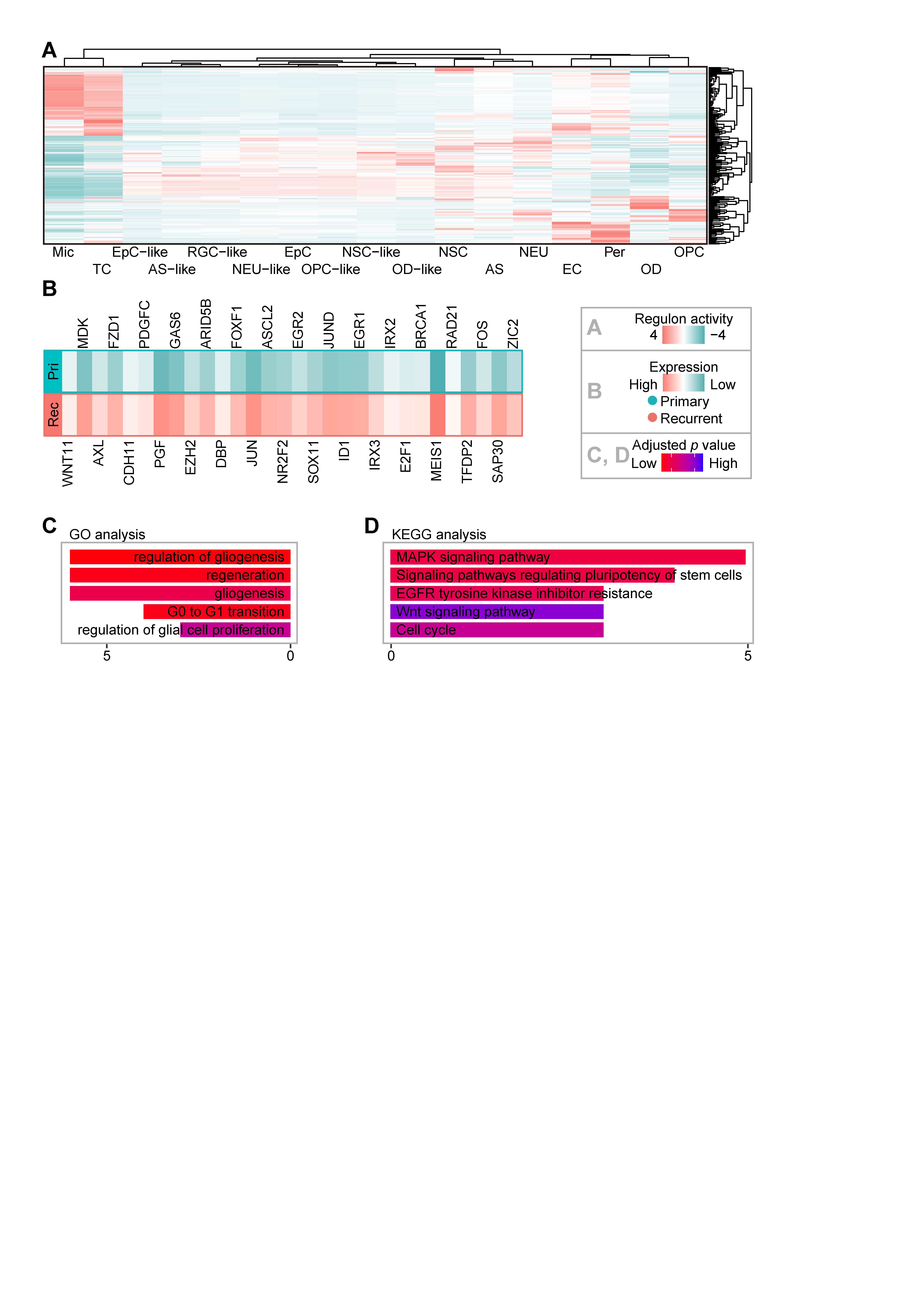

Supplement: Supplementary Figure 9 — Additional regulon analysis of EPN. (A) Gene regulatory networks were inferred by SCENIC and were clustered by cell types (bottom) and regulons (right). (B) List of significantly upregulated genes in recurrent samples from crosstalk and gene regulatory network analysis of NSC-like cells which share the same enriched terms in GO/KEGG analysis. (C, D) Visualization of genes using GO and KEGG enrichment analysis. [file Image_9.jpeg]
